# Frequency of cholecystectomy and associated sociodemographic and clinical risk factors in the ELSA-Brasil study: Frequência de colecistectomia e fatores de risco sociodemográficos e clínicos associados no estudo ELSA-Brasil

**DOI:** 10.1590/1516-3180.2015.0250130216

**Published:** 2015-04-14

**Authors:** Kamila Rafaela Alves, Alessandra Carvalho Goulart, Roberto Marini Ladeira, Ilka Regina Souza de Oliveira, Isabela Martins Benseñor

**Affiliations:** 1 BSc. Postgraduate Student, Department of Medicine, Education and Health, Universidade de São Paulo (USP), São Paulo, SP, Brazil.; 2 MD, PhD. Clinical Epidemiologist and Researcher, Center for Clinical and Epidemiological Research, University Hospital, Universidade de São Paulo (USP), São Paulo, SP, Brazil.; 3 MD, PhD. Attending Physician at Hospital Foundation of the State of Minas Gerais, Epidemiologist in the Municipal Health Department of Belo Horizonte, MG, Brazil and Director of the Longitudinal Study of Adult Health (Estudo Longitudinal de Saúde do Adulto, ELSA-Brasil), Belo Horizonte, MG, Brazil.; 4 MD, PhD. Professor, Department of Radiology, School of Medicine, Universidade de São Paulo (USP), São Paulo, SP, Brazil.; 5 MD, PhD. Professor, Department of Internal Medicine, and Director of Center for Clinical and Epidemiological Research, University Hospital, Universidade de São Paulo (USP), São Paulo, SP, Brazil.

**Keywords:** Cholecystectomy, Risk factors, Obesity, Population characteristics, Brazil

## Abstract

**CONTEXT AND OBJECTIVE:**

There are few data in the literature on the frequency of cholecystectomy in Brazil. The frequency of cholecystectomy and associated risk factors were evaluated in the Brazilian Longitudinal Study of Adult Health (ELSA-Brasil).

**DESIGN AND SETTING:**

Cross-sectional study using baseline data on 5061 participants in São Paulo.

**METHODS:**

The frequency of cholecystectomy and associated risk factors were evaluated over the first two years of follow-up of the study and over the course of life. A multivariate regression analysis was presented: odds ratio (OR) and 95% confidence interval (95% CI).

**RESULTS:**

A total of 4716 individuals (93.2%) with information about cholecystectomy were included. After two years of follow-up, 56 had undergone surgery (1.2%: 1.7% of the women; 0.6% of the men). A total of 188 participants underwent cholecystectomy during their lifetime. The risk factors associated with surgery after the two-year follow-up period were female sex (OR, 2.85; 95% CI, 1.53-5.32), indigenous ethnicity (OR, 2.1; 95% CI, 2.28-15.85) and body mass index (BMI) (OR, 1.10; 95% CI, 1.01-1.19 per 1 kg/m^2^ increase). The risk factors associated over the lifetime were age (OR, 1.03; 95% CI, 1.02-1.05 per one year increase), diabetes (OR, 1.92; 95% CI, 1.34-2.76) and previous bariatric surgery (OR, 5.37; 95% CI, 1.53-18.82). No association was found with parity or fertile age.

**CONCLUSION:**

Female sex and high BMI remained as associated risk factors while parity and fertile age lost significance. New factors such as bariatric surgery and indigenous ethnicity have gained importance in this country.

## RESUMO

**CONTEXTO E OBJETIVO:**

Há escassez de dados na literatura sobre a frequência de colecistectomia no Brasil. Avaliou-se a frequência de colecistectomia e os fatores de risco associados no Estudo Longitudinal de Saúde do Adulto (ELSA-Brasil).

**DESENHO E LOCAL:**

Estudo transversal com dados da linha de base de 5061 participantes em São Paulo.

**MÉTODOS:**

Avaliou-se a frequência de colecistectomia e fatores de risco associados nos dois primeiros anos de seguimento do estudo e ao longo da vida. Apresentou-se regressão logística [razão de chances (RC); intervalo de confiança de 95% (IC 95%)] multivariada.

**RESULTADOS:**

Um total de 4716 (93,2%) indivíduos com informação sobre colecistectomia foi incluído. Após 2 anos de seguimento, 56 participantes tinham sido operados (1,2%: 1,7% nas mulheres; 0,6% nos homens), totalizando 188 participantes com colecistectomia durante a vida. Os fatores de risco associados à cirurgia após dois dois anos de seguimento foram sexo feminino (RC, 2,85; IC 95%, 1,53-5,32), etnia indígena (RC, 2,1; IC 95%, 2,28-15,85) e índice de massa corpórea, IMC (RC, 1,10; IC 95%, 1,01-1,19 por aumento de 1 kg/m^2)^; e, ao longo da vida: idade (RC, 1,03; IC 95%, 1,02-1,05 por um ano de aumento), diabetes (RC, 2,10; IC 95%, 1,34-2,76) e cirurgia bariátrica prévia (RC, 5.37; IC 95%, 1,53-18,82). Não se observou associação com paridade ou idade fértil.

**CONCLUSÃO:**

Sexo feminino e IMC elevado permanecem sendo fatores de risco associados à colecistectomia, mas paridade e idade fértil perderam significância. Novos fatores de risco, como cirurgia bariátrica prévia e etnia indígena, ganharam relevância no país.

## INTRODUCTION

Gallstones have been recognized since antiquity, and have been found in Egyptian mummies.^1^ Today, they are a frequent problem in developed countries, affecting 10 to 15% of the adult population in the United States,^2^ while in Europe, the prevalence ranges from 5.9% to 21.9%.^3^ In Brazil, few studies have evaluated the frequency of gallstones and associated risk factors. Coelho et al*.* ^4^ screened 1000 individuals in two shopping centers in the city of Curitiba, Brazil, using ultrasound, and found that the frequency of gallstones was 6.4%. The direct and indirect costs of this ailment have been progressively increasing over recent years, as a consequence of increased numbers of surgical procedures in recent decades, and especially since the introduction of laparoscopic cholecystectomy in 1989.^5^^,^^6^

Multiple factors are responsible for cholelithiasis. Advancing age is one important risk factor for gallstones, and female sex is also a known risk factor.^2^ Estrogen seems to play a critical role in this increased risk, because pregnancy, high parity and estrogen replacement therapy increase the risk of gallstones.^7^ Obesity is another risk factor for gallstone development, likely caused by increased hepatic secretion of cholesterol.^1^ The risk is especially high among women and increases linearly with increasing body mass index.^8^ Ironically, rapid weight loss is also a risk factor for gallstone development, such that gallstones occur in 25% to 30% of patients who undergo bariatric surgery.^2^ Although most people with gallstones are asymptomatic, about 20% present symptoms at some point and 7% require surgical intervention.^9^

The prevalence of cholecystectomy in the United States is higher among women than among men, and it varies widely according to race.^10^ Maclure et al.^8^ in the Nurses' Health Study reported that the frequency of cholecystectomy was 0.49%,^8^ while Liu et al.^11^ reported a frequency of 1.3% in Taiwan. Except for the study by Coelho et al.,^4^ in which a frequency of 2.9% was reported from ultrasound screening of people in a shopping center in Curitiba, few studies on the epidemiology of cholecystectomy and associated risk factors have been conducted in Brazil.

## OBJECTIVE

The aim of this study was to conduct a cross-sectional investigation of the frequency of cholecystectomy and associated socio-demographic and clinical risk factors using data from all participants in the ELSA-Brasil research center of São Paulo who had baseline information about previous cholecystectomy.

## METHODS

### Study design and population

The Brazilian Longitudinal Study of Adult Health (ELSA-Brasil) is a prospective cohort study designed to investigate the incidence of cardiovascular diseases and diabetes, along with their biological and social determinants. The study originally included 15,105 subjects aged 35-74 years from six cities located in three different regions of Brazil: Belo Horizonte, Porto Alegre, Rio de Janeiro, Salvador, São Paulo and Vitória.^12^^,^^13^^,^^14^ In our analysis, we included baseline (cross-sectional) information from the first examination, which took place between August 2008 and December 2010. Only the participants evaluated at the São Paulo Research Center for whom data about cholecystectomy was available were included in this analysis.

Data were gathered from participants in two phases. The first, which lasted for approximately one hour, consisted of obtaining informed consent and conducting the initial interview at the participant's work site. The second comprised additional interviews and examinations, lasted for approximately six hours and was conducted at the study clinic.^15^ ELSA-Brasil was approved by the Institutional Review Board at the University Hospital of the University of São Paulo, and all participants signed an informed consent statement.

### Cholecystectomy information

Information about the previous cholecystectomy was obtained from the baseline study questionnaire, and was confirmed by means of an ultrasound evaluation that focused on screening for hepatic steatosis, which was also performed at baseline. As part of a multicenter protocol, all of the liver images were obtained in the same position in relation to four anatomical landmarks, one of which was viewing of the gallbladder. More details about the liver ultrasound examination have been published elsewhere.^16^ Participants who did not report having had any previous cholecystectomy or did not undergo hepatic ultrasound were excluded from the analysis.

### Sociodemographic characteristics

We analyzed sociodemographic characteristics such as sex, age (years), self-reported race/skin color (white, mixed, black, East Asian or indigenous), years of schooling (< 11 years, 11-15 years or > 15) years, mean net family income (≤ US$ 1245, US$ 1246-3319 or ≥ US$ 3320), partner status (single or married) and previous history of bariatric surgery (yes or no).^17^ Local currency [Brazilian reais (BRL)] was converted to U.S. dollars (USD) at a rate of BRL 2.00 = USD 1.00 in December 2008.

### Anthropometric and blood pressure measurements

Anthropometric and blood pressure measurements were made on all participants. Weight, height and waist circumference were measured following standard techniques.^18^ Body mass index (BMI) was calculated as weight (in kilograms) divided by squared height (in meters).^19^ Abdominal obesity was defined as a waist circumference > 88 cm among women or > 102 cm among men.^19^ Resting blood pressure was measured three times in a seated position after five minutes of rest, using a validated oscillometric device. The averages of the second and third measurements were taken to be the systolic and diastolic blood pressures in the analyses.^20^

### Cardiovascular risk factors

Hypertension was defined as use of medication to treat hypertension, or systolic blood pressure ≥ 140 mmHg, or diastolic blood pressure ≥ 90 mmHg. Diabetes was defined as a previous medical history of diabetes, or use of medication to treat diabetes, or fasting serum glucose ≥ 126 mg/dl, or two-hour oral glucose tolerance test ≥ 200 mg/dl, or HbA1c levels ≥ 6.5%. Dyslipidemia was defined as LDL-cholesterol > 130 mg/dl or current use of cholesterol-lowering medication. Patients were categorized according to smoking status and alcohol consumption as never, past or current users.

### Psychiatric disorders

Mental diagnoses were assessed by trained interviewers using the validated Portuguese version of the Clinical Interview Schedule - Revised (CIS-R). The CIS-R is a structured interview for diagnosing and measuring non-psychotic psychiatric morbidity in the community. This short and straightforward questionnaire was developed in 1992 by Lewis et al*.* ^21^ to be used specifically within community and primary care. Additionally, diagnoses of specific disorders were obtained by applying algorithms based on the International Classification of Diseases (ICD)-10 diagnostic criteria.^22^

### Surveillance

Surveillance is being conducted through annual telephone interviews, through a second examination four years after the baseline assessment at the ELSA-Brasil research centers, and through linkage to national databases, such as the National Mortality Information System. Annual telephone calls are made to verify the overall state of the participants' health, including new diagnoses, deaths, hospitalizations and emergency department visits over the first two years of follow-up. All diagnoses noted in hospital discharge summaries or hospital records are recorded. Full hospital information is abstracted by trained personnel if the diagnoses include any ICD codes that relate to ELSA-Brasil endpoints.^23^ For this analysis, we used all information about gallbladder procedures from the first two years of follow-up data.

### Statistical analysis

Categorical variables are presented as proportions and were compared using the chi-square test. Continuous variables are presented as means (with standard deviations) and were compared using one-way ANOVA with the Bonferroni post-hoc test.

A logistic regression model was built using sociodemographic characteristics and cardiovascular risk factors as the independent variables, and cholecystectomy after the baseline measurements as the dependent variable. Odds ratio were presented as crude values, adjusted for age and sex, and with multivariate adjustment. For sociodemographic risk factors, we did not present multivariate adjustment because the only socio-demographic characteristics with P < 0.20 in Table 1 were age and sex. For clinical variables, multivariate adjustment was done in accordance with sociodemographic and clinical characteristics with P < 0.20 in Table 1, which were age, sex, body mass index, waist measurement, hypertension, diabetes, dyslipidemia, major depressive disorders and previous bariatric surgery. Other logistic models were produced considering all cases of cholecystectomy (before and after the baseline assessment).

**Table 1:**
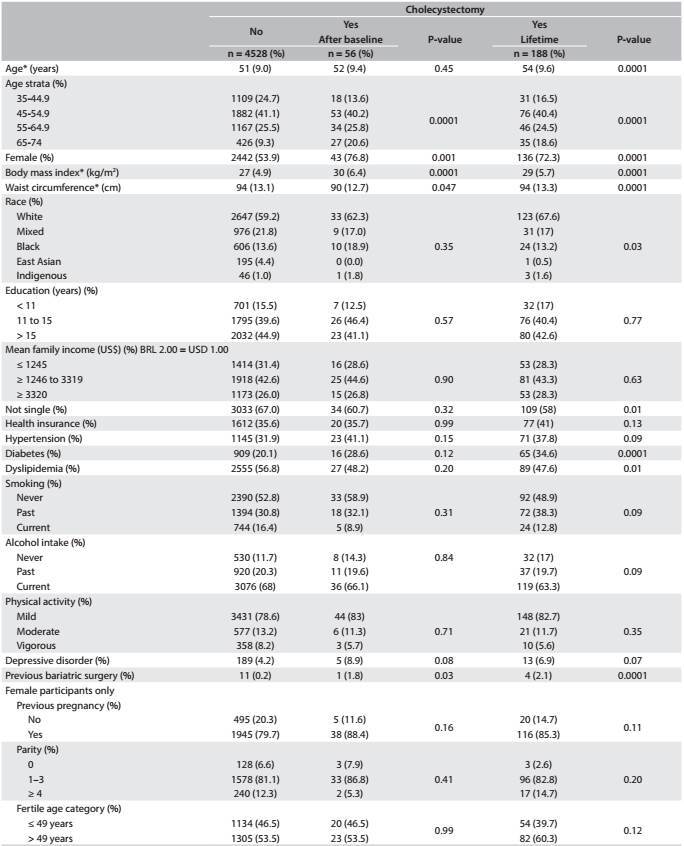
Sociodemographic and clinical characteristics of participants according to cholecystectomy during the first two years of follow-up

*Mean (standard deviation).

A logistic regression model was also built using sociodemographic characteristics and cardiovascular risk factors as the independent variables, and all lifetime cholecystectomy procedures as the dependent variable. For sociodemographic risk factors, multivariate adjustment was done for other sociodemographic characteristics with P < 0.20 in Table 1: age, sex, race, marital status and having private health insurance. For clinical variables, multivariate adjustment was done for all sociodemographic and clinical risk factors with P < 0.20 in Table 1: age, sex, body mass index, waist measurement, marital status, health insurance plan, hypertension, diabetes, dyslipidemia, smoking, alcohol intake, major depressive disorders and bariatric surgery.

The significant level was set at P < 0.05. All the analyses were performed using the SPSS software, version 22.0.

## RESULTS

Overall, there were 5,061 participants (53.9% women) at the São Paulo research center. No information about previous cholecystectomy or liver imaging was available for 345 participants, and these were excluded from the analysis. Consequently, 4,716 participants remained in the study. There were 132 cases of cholecystectomy before enrollment in ELSA-Brasil, 56 cases over the first two years of follow-up and, thus, 188 cases over these individuals' lifetimes.

The prevalence of cholecystectomy before enrollment was 2.8%: 3.6% among women and 1.8% among men. Excluding these cases that underwent cholecystectomy before enrollment, 4,584 participants with full information about cholecystectomy (clinical questionnaire and ultrasound data) over the first two years of follow-up remained in the sample. In this subset, the prevalence of cholecystectomy was 1.2%: 1.7% among women and 0.6% among men. The mean age was 51.4 years (± 8.9) and 76.7% of the total sample were women. The lifetime prevalence of cholecystectomy was 4.0%: 5.3% among women and 2.4% among men.

Table 1 compares several sociodemographic and clinical risk factors among participants who underwent cholecystectomy over the first two years of follow-up, in relation to participants who did not. Additionally, it also includes a comparison of all participants who underwent cholecystectomy at any point during their lifetimes (before or after the baseline) with those who did not.

Participants who underwent cholecystectomy after the baseline were more likely to be women and have higher BMI and waist measurement values, along with higher frequency of previous bariatric surgery, compared with participants who did not undergo surgery (Table 1). In the analysis that included lifetime cases of cholecystectomy (before and after baseline assessment), patients who underwent surgery were older and had higher BMI. The proportions of white and indigenous individuals and of singles and diabetics were higher, while the proportion of the patients with dyslipidemia was lower than the proportion who did not undergo cholecystectomy.

^Tab^ le 2 presents logistic models for sociodemographic and clinical risk factors for all participants who underwent cholecystectomy during the first two years of follow-up. After multivariate adjustment, we found that there were positive associations with female sex (OR, 2.85; 95% confidence interval, 95% CI, 1.53-5.32) and indigenous ethnicity (OR, 2.10; 95% CI, 2.28-15.85). We also detected a positive OR of 1.10 (95% CI, 1.01-1.19) per 1 kg/m^2^ increase in BMI, which remained significant after multivariate adjustment.

**Table 2:**
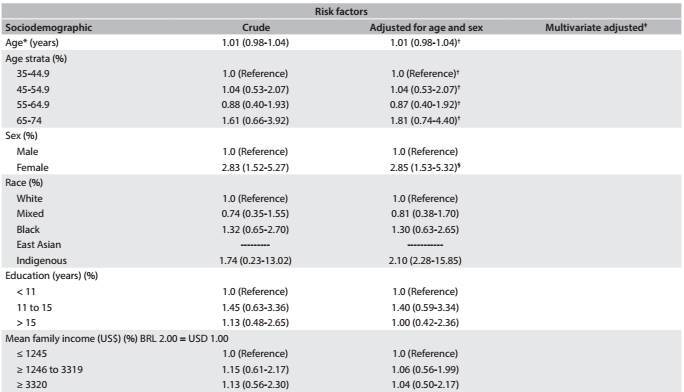
Odds ratio (OR) and 95% confidence intervals (95% CI) showing sociodemographic and clinical risk factors associated with cholecystectomy during the first two years after baseline examination among ELSA-Brasil participants at the São Paulo research center

**Table 2:**
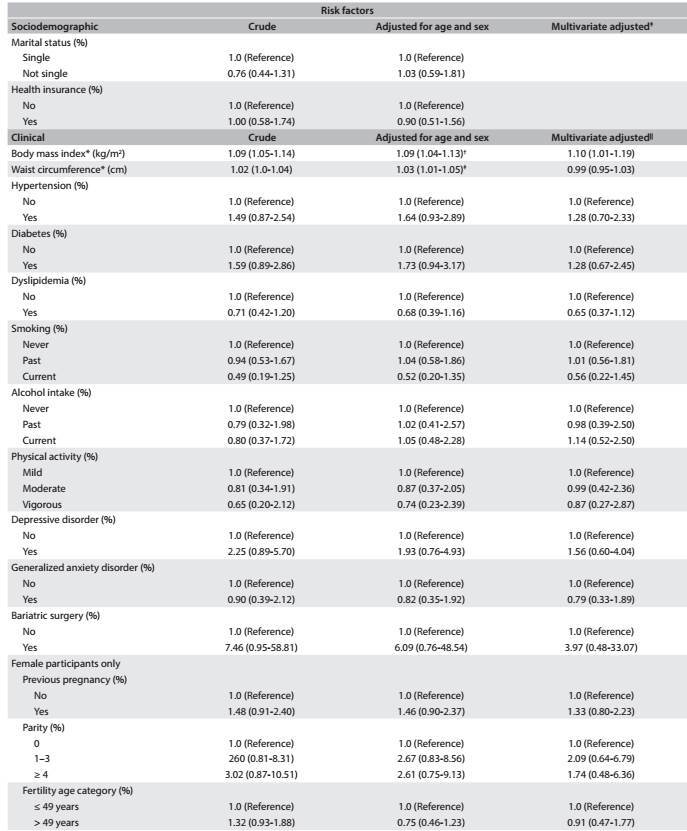
Continues...

*Mean (standard deviation); ^†^Adjusted for sex; ^‡^Multivariate adjustment was not presented because only age and sex had P < 0.20; ^§^Adjusted for age; ^||^Multivariate adjustment for age, sex, race, education, mean net family income, marital status, health insurance, BMI, waist circumference, hypertension, diabetes, dyslipidemia, smoking, alcohol intake, physical activity, depressive disorder and bariatric surgery.

^Tab^ le 3 shows the same associations, taking into consideration all the participants who underwent cholecystectomy (before and after the baseline, combined). After multivariate adjustment, we found that there were positive associations with age (for each one year increase in age: OR, 1.03; 95% CI, 1.02-1.05), female sex (OR, 2.35; 95% CI, 1.65-3.33), diabetes (OR, 1.92; 95% CI, 1.34-2.76) and previous bariatric surgery (OR, 5.37; 95% CI, 1.53-18.82), which remained significant after multivariate adjustment. East Asian ethnicity (OR, 0.09; 95% CI, 0.01-0.65) and dyslipidemia (OR, 0.62; 95% CI, 0.45-0.85) were protective factors against cholecystectomy. We did not find that previous pregnancy, parity or the age stratum from 35 to 49 years (stratum of fertile age) showed any association with cholecystectomy in this subset of the ELSA-Brasil participants.

**Table 3:**
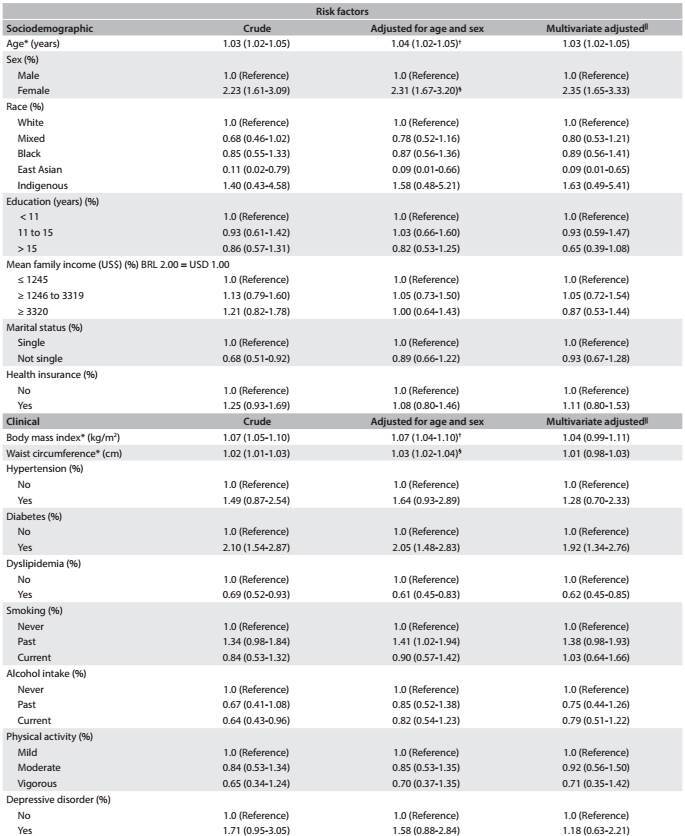
Odds ratio (OR) and 95% confidence intervals (95% CI) showing sociodemographic and clinical risk factors associated with lifetime occurrence of cholecystectomy among ELSA-Brasil participants at the São Paulo research center

**Table 3:**
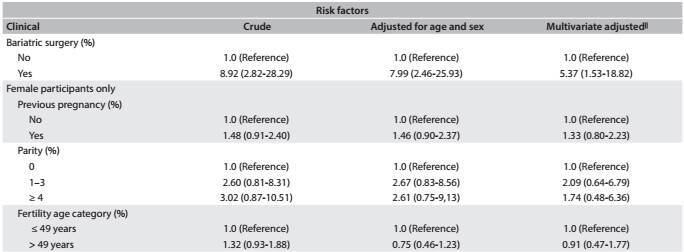
Continues...

*Mean (standard deviation); ^†^Adjusted for sex; ^‡^Multivariate adjustment for all sociodemographic variables; ^§^Adjusted for age; ^||^Multivariate adjustment for age, sex, race, education, mean net family income, marital status, health insurance, BMI, waist circumference, hypertension, diabetes, dyslipidemia, smoking, alcohol intake, physical activity, depressive disorder, generalized anxiety disorder and bariatric surgery.

Table 4 describes the frequencies of elective and laparoscopic surgery according to sex. Most surgeries were laparoscopic and performed as elective procedures for women and as an emergency for men. However, these differences about the number of elective surgery according to sex were not statistically significant.

**Table 4:**
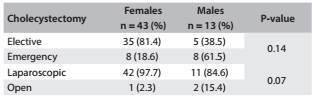
Type of cholecystectomy during the first two years of follow-up according to sex.

## DISCUSSION

In summary the prevalence of cholecystectomy in the baseline examination was 2.8% (3.6% among women and 1.8% among men). The frequency of cholecystectomy over the first two years of follow-up was 1.2% (1.7% among women and 0.6% among men). The lifetime prevalence of cholecystectomy was 4% (5.3% among women and 2.4% among men) We found that female sex, indigenous ethnicity and high BMI values were associated with cholecystectomy performed during the first two years of follow-up. For lifetime cholecystectomy (cholecystectomy before and after follow-up), positive associations were found with age, female sex, diabetes and bariatric surgery. East Asian ethnicity and dyslipidemia were protective factors against cholecystectomy among the ELSA-Brasil participants. No association was found between cholecystectomy and previously known risk factors for women such as previous pregnancy, parity or age within the fertile stratum (35 to 49 years), with regard to either lifetime cholecystectomy or its occurrence during the first two years of follow-up.

Some studies conducted in other countries have investigated the frequency of cholecystectomy in apparently healthy populations. A cross-sectional study in Taiwan evaluated 2,386 healthy adults and reported that the prevalence of cholecystectomy was 1.3%.^11^ A cross-sectional analysis within the German Study of Health in Pomerania (SHIP) cohort evaluated 4,202 subjects aged 20-79 years and found that the prevalence of previous cholecystectomy before enrollment in the study cohort was 11.1%.^24^ In Italy, Barbara et al.^25^ evaluated 1,911 people aged 18 to 65 in the township of Sirmione and found that the prevalence of previous cholecystectomy was 4.1%. A German study with 2,147 participants aged 10 to 65 years found a prevalence of 3.9%.^26^ Only one Brazilian study evaluated the frequency of cholecystectomy, in a sample of 1,000 apparently healthy men (479) and women (521), and it reported a frequency of 2.9%,^4^ i.e. similar to the frequency of cholecystectomy in our sample before enrollment in ELSA-Brasil. The results from the present study were similar to those of this previous Brazilian study, but were higher than those of the Taiwan study and lower than the prevalence in Europe, especially compared with the SHIP study, which yielded significantly higher prevalence than the other studies. Considering the lifetime prevalence of cholecystectomy, our results are similar to the European data, except for the SHIP study.

Although data on the prevalence of cholecystectomy worldwide is available, data on its incidence is very scarce. Maclure et al.^8^ evaluated the incidence of cholecystectomy in women in the Nurses' Health Study and found 433 new cases in a 4-year follow-up among 88,837 women aged 34 to 59 years.^8^ As we did not yet have the estimate of person-years from the ELSA-Brasil survey, in this present study we chose to use logistic models and the frequency of cholecystectomy over the first two years of follow-up, rather than the Cox proportional hazards model, to calculate the incidence of cholecystectomy in the sample.

One important limitation in our analysis is that we did not have information about the presence of gallstones, since in the ELSA-Brasil survey, we did not perform complete ultrasound scans with decubitus changes in order to look for gallstones. Our main objective was to evaluate the presence of signs of fatty liver disease in the sample, and our protocol focused on including the gallbladder, whenever possible, as one anatomical landmark in the liver images acquired. In this way, we gathered information on whether the gallbladder was present or not when we acquired the image of the right liver lobe, in the same position for all participants who underwent ultrasound measurements.

As stated previously by Pedersen et al.,^27^ the prevalence of cholecystectomy does not exactly reflect the prevalence of gallstones. There are also other factors involved, such as increased frequency of symptomatic gallstones, better access to health services, availability of ultrasound examination or a lowered clinical threshold for gallbladder surgery, which could affect this relationship.

Another limitation of the present study in relation to calculating the incidence of cholecystectomy was that the length of follow-up was too short to evaluate the risk of new cases of cholecystectomy based on risk factors identified at the baseline examination.

Our results showed that the main risk factors associated with cholecystectomy after the baseline examination of this study were female sex, indigenous ethnicity and high BMI. On analyzing all the cases of cholecystectomy together, we also found associations with age, higher frequency of being diabetic and previous bariatric surgery.

Aging is associated with gallstones in all racial groups,^28^ and in both sexes.^25^^,^^29^ Most studies also showed higher frequencies of gallstones and cholecystectomy among women than among men.^25^^,^^29^ Everhart et al.^10^ used data from the NHANES III study to evaluate the frequencies of gallstones and surgery to treat gallstones among men and women according to race. They found that among men, the frequency of surgery was around 25 to 35% of the total frequency of gallstones, while among women, this frequency was around 40 to 50%, and neither of these results was related to race. However in the same sample, among Mexican-Americans, more than 50% of the women underwent surgery.^10^ A possible explanation for the condition that women are more affected by gallstones than men may be related to the use of estrogen. It has been demonstrated that parity is an important risk factor for gallbladder disease.^30^

Interestingly, in our sample, we did not find any relationship between cholecystectomy and previous pregnancy, parity or the fertile period (from 35 to 49 years of age among women). It is likely that these risk factors have lost their importance over recent years. Fertility rates are progressively declining in Brazil, especially in the state of São Paulo. It is possible that a progressive decline in fertility rates could decrease the impact of parity on gallbladder disease and cholecystectomy in this state. However, Walcher et al.^26^ did not find any association between parity and cholecystectomy in Germany in 2005.

Even the association with age does not seem as important now as it once was. In relation to the lifetime prevalence of cholecystectomy, there was a positive association, but if the analysis was restricted to the first two years of follow-up, we found that there was no association between cholecystectomy and aging. However, it is possible that we did not have sufficient statistical power because of the low number of cases.

Ethnicity is another very important risk factor. The highest prevalence of cholelithiasis occurs among North American Indians, such that 73% of female Pima Indians over the age of 30 years are affected,^31^ with high rates among other American and Canadian Indians.^32^ Although few studies have been published, South American Indians have a similarly high prevalence of gallbladder disease. In Chile, the native Mapuche Indians demonstrate this elevated occurrence: 49.4% among women and 12.6% among men (> 60% among women in their fifties). The frequency is lower among Chilean Hispanics with lesser degrees of American Indian admixture: 36.7% among women and 13.1% among men.^33^ The prevalence of gallstones among Mexican-Americans is also a direct function of the degree of Amerindian admixture. White Americans have somewhat lower prevalence.^10^ This is the first Brazilian study to show an association between indigenous ethnicity and higher risk of cholecystectomy, thus showing that this high prevalence among Indians is also valid for Brazil. In our sample, we also observed that East Asian ethnicity was a protective factor against cholecystectomy. This is corroborated by the study by Liu et al.^11^ in Taiwan, which found a very low prevalence of 1.3%.

It is interesting that presence of diabetes was also a significant factor when considering all the cases of cholecystectomy. There is no clear explanation in the literature for a higher frequency of gallstones among diabetics. However, gallbladder disease and diabetes have a very important common risk factor, obesity,^34^ and its effect is stronger among women than among men. Diabetes, abdominal obesity and gallstones appear to be linked through metabolic syndrome.^35^^,^^36^ Liu et al.^11^ also reported an association between cholecystectomy and type 2 diabetes.

In our study, dyslipidemia was protective against gallbladder surgery. This is probably because our definition of dyslipidemia included LDL-cholesterol < 130 mg/dl and/or use of statins. Use of statins was described in a previous study as a protective factor against cholelithiasis.^37^ Interestingly, we found that the association between depressive disorders and cholecystectomy showed borderline significance (P = 0.08) among participants undergoing surgery after baseline, and P = 0.07 for all participants who had cholecystectomy. It is possible that we did not have sufficient statistical power for the analysis, but this can be investigated in the future with a longer follow-up period.

Previous bariatric surgery is a factor associated with chole-cystectomy that has emerged more recently and is gaining importance, due to the increasing frequency of both obesity and bariatric surgery in Brazil and worldwide. Rapid weight loss on low-calorie diets or after bariatric surgery is a major risk factor for cholesterol gallstone formation.^1^ Sludge and gallstones develop following bariatric surgery on extremely obese individuals in as many as 25-35% of the cases,^2^ usually during the first six weeks after surgery, when the weight loss is most profound.^38^

In the present study, laparoscopic surgery was the main method used for cholecystectomy and most of the operations were elective procedures. Laparoscopic cholecystectomy has been the current surgical standard for most diseases of the gallbladder over the past three decades.^39^^,^^40^ It was no surprise that we found results similar to previous findings^39^^,^^40^ in a sample with better access to health services than the general population in Brazil. A study at the University of Michigan Medical Center investigated 772 patients who underwent cholecystectomy and found that the frequency of laparoscopic surgery (77.8%) was higher than that of open cholecystectomy (5.7%).^41^ A study using the database of the Health Care Utilization Project - Nationwide Inpatient Sample (HCUP-NIS) analyzed 358,091 patients who underwent cholecystectomy procedures from 1999 to 2006. The authors of this study reported that laparoscopic surgery was performed more frequently than open cholecystectomy, and that there was a gradual increase in laparoscopic surgery across all age groups (≥ 18 years) during this period.^42^

Our study has some strengths. It had a large sample of apparently healthy men and women with detailed information about the risk factors that may be associated with cholecystectomy. Only one Brazilian study evaluated the frequency of cholecystectomy worldwide assessed samples that did not have previous complaints associated with gallbladder stones.^4^ Our study also has some limitations. It was a cross-sectional analysis and therefore only allowed assessment of associations, rather than causality. We only had information about the frequency of cholecystectomy, without any information about cholelithiasis, unlike other studies that evaluated both of these factors.

## CONCLUSIONS

In conclusion, the frequency of lifetime cholecystectomy was similar to values that have previously been published in studies worldwide. As in other countries, most of the surgeries were laparoscopic procedures. Female sex and high BMI values continue to be risk factors, but others, such as parity and fertile age, lost significance, while new factors such as bariatric surgery and indigenous Brazilian ethnicity are gaining importance.
